# Modulation of *yellow* expression contributes to thermal plasticity of female abdominal pigmentation in *Drosophila melanogaster*

**DOI:** 10.1038/srep43370

**Published:** 2017-02-23

**Authors:** Jean-Michel Gibert, Emmanuèle Mouchel-Vielh, Frédérique Peronnet

**Affiliations:** 1 Team “Epigenetic control of developmental homeostasis and plasticity”, Sorbonne Universités, UPMC Univ Paris 06, CNRS, Biologie du Développement Paris Seine-Institut de Biologie Paris Seine (LBD-IBPS), 75005 Paris, France

## Abstract

Phenotypic plasticity describes the ability of a given genotype to produce distinct phenotypes in different environments. We use the temperature sensitivity of abdominal pigmentation in *Drosophila melanogaster* females as a model to analyse the effect of the environment on development. We reported previously that thermal plasticity of abdominal pigmentation in females involves the pigmentation gene *tan (t)*. However, the expression of the pigmentation gene *yellow (y*) was also modulated by temperature in the abdominal epidermis of pharate females. We investigate here the contribution of *y* to female abdominal pigmentation plasticity. First, we show that *y* is required for the production of black Dopamine-melanin. Then, using *in situ* hybridization, we show that the expression of *y* is strongly modulated by temperature in the abdominal epidermis of pharate females but not in bristles. Interestingly, these two expression patterns are known to be controlled by distinct enhancers. However, the activity of the *y-wing-body* epidermal enhancer only partially mediates the effect of temperature suggesting that additional regulatory sequences are involved. In addition, we show that *y* and *t* co-expression is needed to induce strong black pigmentation indicating that *y* contributes to female abdominal pigmentation plasticity.

Phenotypic plasticity is “the property of a given genotype to produce different phenotypes in response to distinct environmental conditions”^1^. This is frequently observed in the wild^2^,^3^, and has also major implications in medicine and agricultural sciences. Furthermore, it is thought to facilitate evolution^4^,^5^,^6^,^7^. Indeed, cryptic genetic variations can be revealed by environmental changes. These variations can subsequently be selected, thus contributing to evolution. Although phenotypic plasticity has been described in many animals and plants, its molecular bases are only beginning to be understood. Strikingly, environmental conditions can dramatically affect the transcriptome and several studies have identified modulated genes involved in the plasticity of particular morphological traits^8^,^9^,^10^,^11^.

Abdominal pigmentation in Drosophilids represents an attractive model to dissect the molecular bases of phenotypic plasticity as it is sensitive to temperature in many species^12^. Abdominal pigmentation of *Drosophila melanogaster* females is darker when they develop at low temperature^13^. This is particularly pronounced in posterior abdominal segments (A5, A6 and A7) (Fig. 1). Plasticity of abdominal pigmentation is likely to have functional consequences as abdominal pigmentation has been linked to thermoregulation and resistance to UV, pathogens or parasites^13^,^14^,^15^,^16^. Abdominal pigmentation was also associated to resistance to desiccation^17^, but this was not confirmed in a recent study^18^. Abdominal pigmentation differs between males and females in several *Drosophila* species and has been used as a model to dissect the genetic bases of sexual dimorphism^19^,^20^. Furthermore, as abdominal pigmentation is highly evolvable^21^, it has been investigated to study the molecular bases of morphological variation within or between species^22^,^23^,^24^,^25^,^26^,^27^,^28^,^29^. The genes involved in *Drosophila* abdominal pigmentation are relatively well known, in particular those encoding the enzymes required for the synthesis of cuticle pigments^30^,^31^,^32^,^33^ (Fig. 2). We reported recently that the thermal plasticity of female abdominal pigmentation in *Drosophila melanogaster* involves transcriptional modulation of the pigmentation gene *tan (t*)^34^. This gene encodes a hydrolase implicated in the production of melanin^35^ (Fig. 2). *t* is seven times more expressed at 18 °C than at 29 °C in the posterior abdominal epidermis of young adult females. Furthermore, genetic experiments showed that *t* plays an essential role in the plasticity of female abdominal pigmentation. However, we also reported that the expression of the pigmentation genes *yellow (y), ebony (e), Dopa-decarboxylase (Ddc)* and *black (b)* was modulated in pharates (late pupae) but more moderately (less than two fold). This modulation of their expression by temperature is consistent with a darker pigmentation at low temperature and likely explains the residual pigmentation plasticity observed in *t* loss-of-function mutants.

We investigate here the contribution of *y* to female abdominal pigmentation plasticity. The *y* gene has been known for a hundred years as it was among the first described *Drosophila melanogaster* mutants^36^. It is required for the production of black melanin and in the absence of *y*, black melanin is replaced by brown melanin (see Fig. 2)^37^. In *Drosophila melanogaster, y* is sex-specifically regulated in the posterior abdomen in correlation with the sexual dimorphism of the melanic pattern observed in adults^25^. Furthermore, the evolution of wing or abdominal pigmentation patterns between *Drosophila* species correlates with modifications of *y* spatial expression^27^,^37^,^38^,^39^,^40^,^41^. We show here that temperature modulates the spatial expression of *y* in the abdominal epidermis of pharate females in correlation with the modulation of cuticle pigmentation observed in adults. By contrast, *y* expression associated with bristles is not modulated by temperature. *y* is known to be required but not sufficient for black melanin production^37^. Our data indicate that this black melanin is Dopamine-melanin and not Dopa-melanin. Furthermore, we show that combined over-expression of *y* and *t* at 29 °C is necessary and sufficient to reproduce the black phenotype observed at 18 °C. Thus, the stronger expression of *y* at 18 °C contributes to thermal plasticity of female abdominal pigmentation.

## Results

### *yellow* is required for the production of Dopamine-melanin

**Figure 2:**
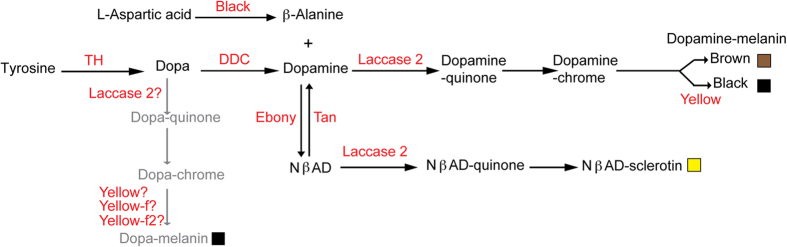
Synthesis pathway of cuticular pigments. Enzymes are indicated in red. In this representation, black melanin is considered as Dopamine-melanin (see text for experimental justification) and the alternative pathway mentioned by several authors leading to Dopa-melanin is represented in grey. NβAD: N-β-Alanine-Dopamine.

**Figure 3:**
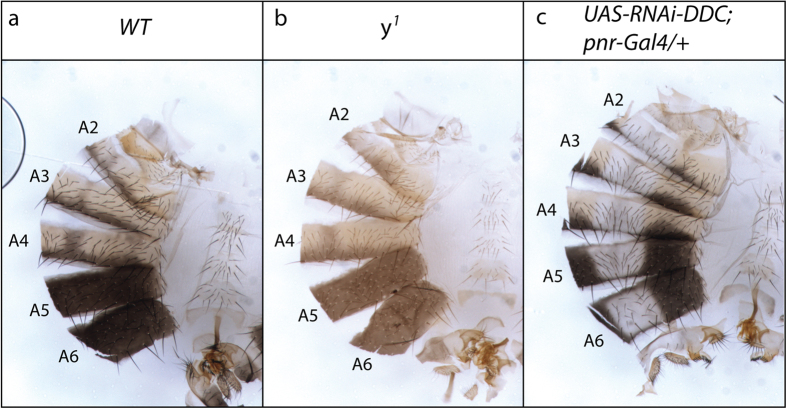
*y* is required for the production of Dopamine-melanin. The posterior abdominal segments of *Drosophila melanogaster* males (A5 and A6) are black (**a**). In *y*^*1*^ mutant males, the black pigment is lost and only brown pigment is visible (**b**). Dorsal down-regulation of *Ddc* using *pnr-*Gal4 to drive an UAS*-RNAi-Ddc* transgene in males leads to loss of both black and brown pigments (**c**).

There is a relative uncertainty in the literature on the nature of the black pigment found in the abdominal cuticle of *Drosophila* when *y* is functional. Indeed, as Yellow is related to two other enzymes, Yellow-f and Yellow-f2, which can use as substrate Dopa-chrome with a higher efficiency than Dopamine-chrome^33^, some authors have proposed that the black pigment in abdominal cuticle was Dopa-melanin produced from Dopa^21^,^22^ (Fig. 2). However, incubation of abdominal cuticles or wings of unpigmented pharates with Dopamine is sufficient to produce black pigment, which suggests that this black pigment is produced from Dopamine and is therefore Dopamine-melanin^30^,^32^. We reasoned that if the black pigment of cuticle were Dopa-melanin, the inactivation of *Ddc* should leave it unaffected (Fig. 2). On the opposite, if black pigment were Dopamine-melanin, the inactivation of *Ddc* should lead to loss of black and brown pigments. We therefore took advantage of the Gal4/UAS system to down-regulate the expression of *Ddc* in the dorsal abdominal epidermis of males using a *Ddc* RNAi transgene^42^ and the *pannier-Gal4* driver (*pnr-Gal4*) (Fig. 3). As *pnr* is expressed only in the dorso-central region of the body^43^, the lateral regions can be used as internal controls. We chose males because of their characteristic black pigmentation in abdominal segments A5 and A6 (Fig. 3a), which is well known to require *y* (Fig. 3b). We observed that *Ddc* down-regulation leads to a complete loss of black and brown pigments (Fig. 3c). We therefore concluded that the black pigment is Dopamine-melanin and not Dopa-melanin.

### Temperature modulates the spatio-temporal expression of *y* in abdominal epidermis

**Figure 4:**
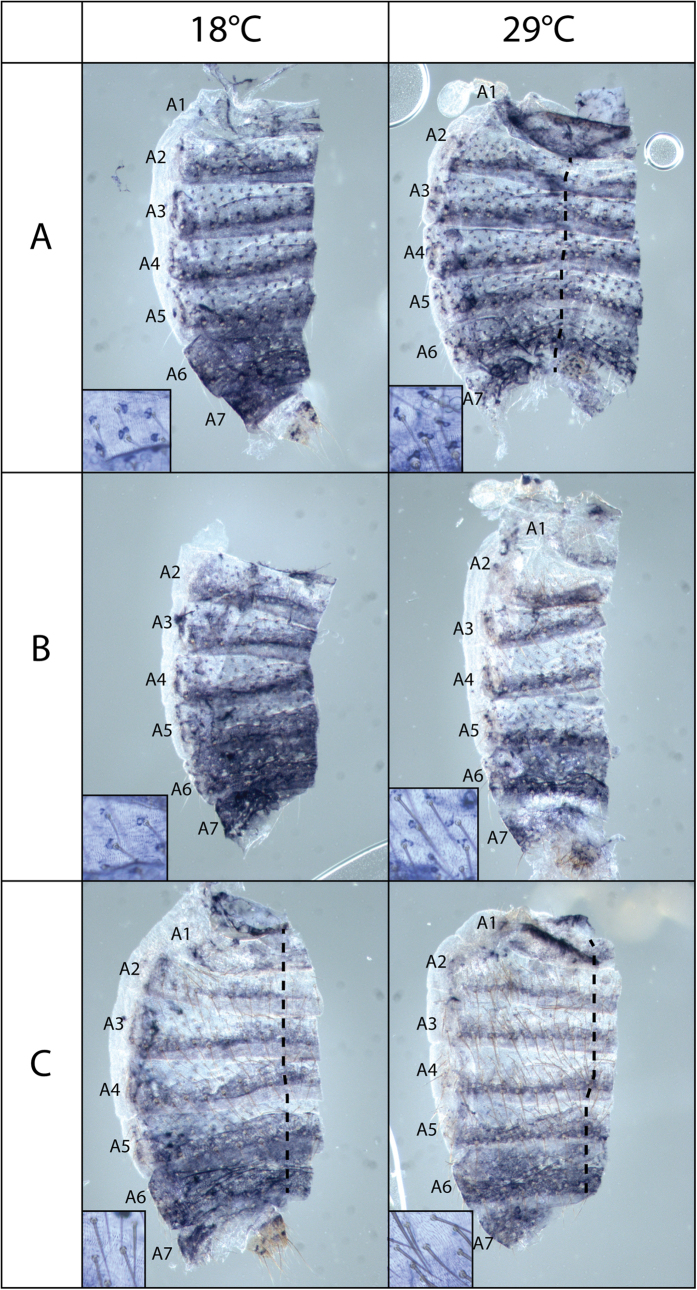
Temperature modulates the spatio-temporal expression of *y* in female pharates. *y in situ* hybridization in the abdomen of *w*^*1118*^ female pharates (stage **A**,**B** and **C**) grown at 18 °C or 29 °C. Cuticles were cut next to the dorsal midline. When the cut was made more distantly, the dorsal midline is indicated by a dashed line in the preparations. Small frames in each picture show the staining associated with bristles at a higher magnification.

We showed previously by RT-qPCR that *y* expression is modulated by temperature in the epidermis of abdominal segments A5, A6 and A7 in female pharates (1.97 fold more expressed at 18 °C than at 29 °C)^34^. In order to analyse the spatial expression of *y,* we performed *in situ* hybridization of female pharates grown at 18 °C or 29 °C. We could distinguish three stages of *y* expression (A, B and C) based on the degree of maturation of abdominal bristles (Fig. 4). These stages correspond approximately to a transition from stage P11(i) to stage P12(ii) as described by Bainbridge and Bownes with morphological markers at 25 °C^44^.

In stage A pharates, two cells at the base of bristles expressed *y*. This expression had a similar intensity when pharates were raised at 18 °C and at 29 °C. These two cells are likely to be the socket and the shaft, the only pigmented cells of the bristle organ. In addition, *y* was expressed in the posterior region of each tergite in segments A2 to A6. This expression was much broader and stronger in pharates grown at 18 °C compared to 29 °C. In A6, *y* was expressed in the whole tergite at 18 °C, and only in the posterior region of the tergite at 29 °C. In A7, at 18 °C, the whole tergite expressed *y* at a high level, whereas it was much weaker at 29 °C.

In stage B pharates, *y* expression was reduced in the socket and the shaft, while the bristle began to be pigmented. Furthermore, *y* was still more expressed in the abdominal epidermis of pharates grown at 18 °C than at 29 °C.

In stage C pharates, *y* was no longer expressed at the base of bristles and the bristles were almost fully pigmented. Furthermore, its overall expression in tergites was reduced compared to stage B and more similar between pharates grown at 18 °C and 29 °C.

Therefore, these data showed that temperature modulates the expression of *y* in stages A and B pharates, *i.e.* when its expression is the highest (corresponding approximately to stages P11(i) to P12(i)^44^).

### The temperature sensitivity of *y* is only partially mediated by the *y-wing-body* enhancer

**Figure 5:**
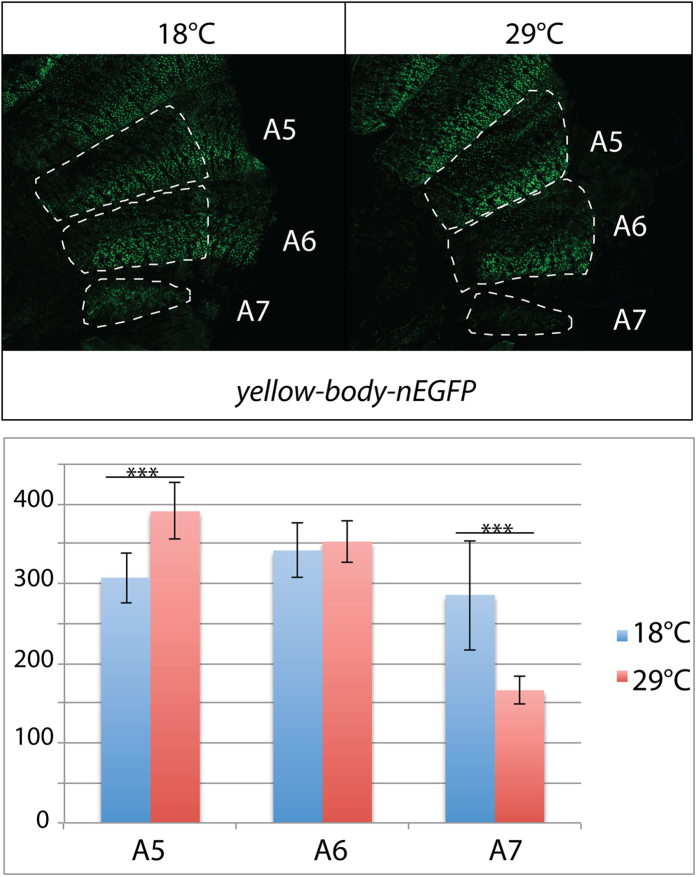
Effect of temperature on the activity of *y* wing-body-enhancer. Top: abdominal epidermis of *y-wing-body–nEGFP* transgenic females grown at 18 °C or 29 °C. The hemi-tergites A5, A6 and A7 used for nEGFP quantification have been circled with white dashed lines. Representative pictures are shown. Bottom: quantification of nEGFP. ***p < 0.001 (n = 10 for each temperature). Exact p-values: A5 p = 2.73E-5; A6 p = 0.43; A7 p = 3.24E-4.

Expression of *y* in wings and abdominal epidermis was shown to be controlled by an enhancer called *y-wing-body* located within the 3 kb upstream of *y* transcription start site^25^. We used the transgenic line *y-wing-body*-*nEGFP* (nuclear Enhanced Green Fluorescent Protein) to test the effect of temperature on the activity of this enhancer (Fig. 5). Unexpectedly, nEGFP was more expressed at 29 °C than at 18 °C in A5. Furthermore, no difference was detected in A6. However, in A7, nEGFP was more expressed at 18 °C than at 29 °C (1.72 fold). In conclusion, nEGFP expression mimicked *y* expression only in A7.

### *y* and *t* co-overexpression at 29 °C is sufficient to reproduce the phenotype observed at 18 °C

**Figure 6:**
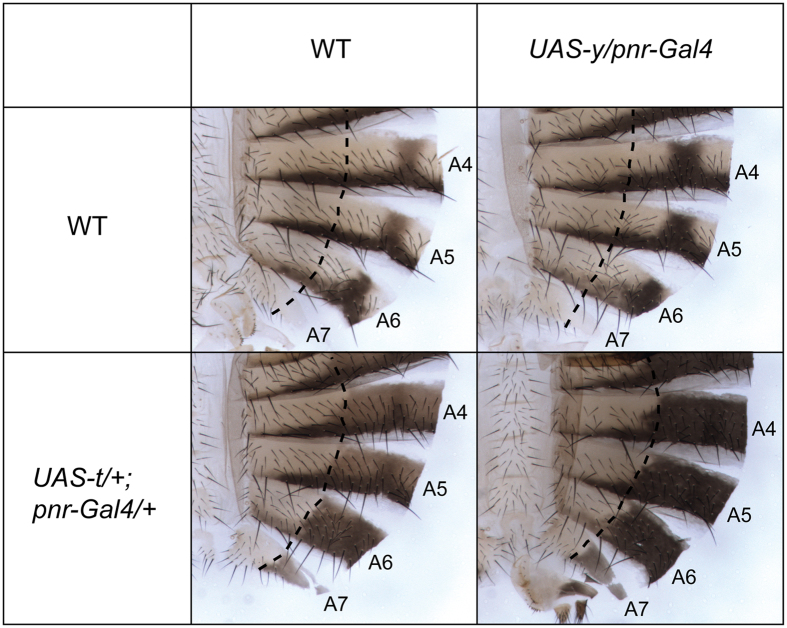
*y* and *t* combined over-expression at 29 °C is sufficient to mimic the pigmentation phenotype observed at 18 °C. Abdominal cuticle phenotypes of wild-type females, females over-expressing *y (UAS-y/pnr-Gal4*), females over-expressing *t (UAS-t/*+*; pnr-Gal4/*+) and females over-expressing both *y* and *t (UAS-t/*+*; UAS-y/pnr-Gal4*) grown at 29 °C. The domain of *pnr* expression is located to the right of the dashed line. Over-expression of *y* is not sufficient to induce the production of black pigment. Over-expression of *t* induces dark pigmentation but pigmentation in the anterior part of the tergites (ectopic pigmentation induced by *pnr*) is lighter than in the posterior part of the tergites (orthotopic plus ectopic pigmentation). By contrast, combined over-expression of *t* and *y* leads to strong and uniform black pigmentation.

*y* is required but not sufficient for production of black pigment^37^. Indeed, *y* gain- of-function must be combined to *e* down-regulation or *t* up-regulation to induce a fully black pigmentation^24^,^37^. In order to test whether the strong expression of *y* an *t* is sufficient to explain the black pigmentation observed at 18 °C, we increased their expression in abdominal epidermis at 29 °C to mimic the effect of lower temperature. We compared the cuticles of wild-type females and females over-expressing either *y (pnr-Gal4/UAS-y*), *t (UAS-t/*+*; pnr-Gal4/*+) or both *y* and *t (UAS-t/*+*; UAS-y/pnr-Gal4)* at 29 °C (Fig. 6). As previously described, *y* over-expression did not change pigmentation whereas *t* over-expression induced dark pigmentation in the anterior region of the tergites^22^,^37^. However, careful examination revealed that this ectopic pigmentation was not as dark as the normal pigmentation in the posterior region of the tergites. This was more visible in A4 and A5 segments. By contrast, when both *y* and *t* were over-expressed in the dorsal region of the abdomen, the anterior region of the tergites was as black as the posterior border of the tergites. Thus, these data show that *y* and *t* combined over-expression at 29 °C is necessary and sufficient to reproduce the pigmentation phenotype observed at low temperature.

## Discussion

In *Drosophila, y* is required for the production of black pigment that we demonstrate here to be Dopamine-melanin. This identification of *Drosophila* black pigment as Dopamine-melanin is in agreement with recent studies analysing the effect of RNAi against *Ddc* on melanin production in other insects^45^,^46^. The diversification of wing or abdomen pigmentation patterns during *Drosophila* evolution was shown to correlate with the modification of *y* expression through changes of either *cis-*regulatory sequences or *trans*-regulator(s)^27^,^37^,^38^,^39^,^40^,^41^. We provide here a detailed description of *y* expression in the abdomen of female pharates grown at 18 °C and 29 °C. We show that temperature modulates the spatio-temporal expression of *y* in the abdominal epidermis of pharates in correlation with the pigmentation pattern observed in adults. Interestingly, expression of *y* in bristle cells is not modulated by temperature. The expression of *y* in bristle cells and in abdominal epidermis is known to be regulated by distinct *cis-*regulatory sequences. In bristle cells, *y* is controlled by an enhancer located in the large intron, whereas in abdominal epidermis it is controlled by the *y-wing-body* enhancer^25^,^47^. The regulatory sequences that control *y* expression in bristle cells are likely to be very constrained as they are found in this intron in 6 *Drosophila* species, whereas those that control *y* expression in the abdominal epidermis are more flexible as they have changed location between these species^47^. Thus, the evolutionary flexibility of *y* regulatory sequences and the temperature sensitivity of *y* expression might be related.

Our results show that temperature sensitive expression of *y* in the epidermis of A7 is mediated at least partly by the *y-wing-body* enhancer, demonstrating that regulation of *y* by temperature occurs at the level of transcription. However, this enhancer does not confer temperature sensitivity to *y* expression in the other segments. The fact that we observed the plastic response in A7 (nEGFP signal 1.72 fold stronger at 18 °C than 29 °C, similarly as the 1.97 fold difference of expression of *y* previously measured by RT-qPCR) is not in favour of a lack of sensitivity of the method. We cannot exclude that genomic sequences flanking the insertion site of the transgene influence the activity of the enhancer and reduce the effect of temperature on its activity specifically in A5 and A6. However, this could also indicate that other regulatory sequences than the *y-wing-body* enhancer, located in the vicinity of the endogenous *y* locus, participate or condition the sensitivity of *y* transcription to temperature. Interestingly, *y* maps close to the telomere of the X chromosome, a region with particular properties such as reduced recombination rate and low genetic variation^48^. Furthermore, *y* is juxtaposed to a binding site for the insulator protein Su(Hw) which separates it from the *achaete-scute* complex^49^. The sensitivity of *y* transcription to temperature changes might therefore be influenced by telomeric chromatin and/or by this insulator. Deciphering these effects would require to manipulate the endogenous *y* locus. Alternatively, upstream regulators of *y* might be themselves temperature sensitive. It would be interesting to identify such factors and find out whether they also regulate other pigmentation genes notably *t* or *e*. Indeed, a recent study identified several transcription factors involved in the regulation (at least indirect) of both *t* and *e*^50^. Lastly, temperature sensitivity of an enhancer was shown to result from a particular arrangement of binding sites for transcriptional regulators^51^. Hence, another hypothesis would be that the architecture of the *y-wing-body* enhancer itself explains its temperature sensitive activity.

Down-regulation of *e* is required to see the effect of *y* over-expression indicating that *y* is not sufficient for black pigment production^37^. We have shown previously that *e* is slightly less expressed at 18 °C than at 29 °C in the anterior abdominal segments of young adult females^34^. Thus, the broader expression of *y* in combination with the lower expression of *e* might contribute to the weak but distinguishable enhancement of black pigment production at 18 °C in anterior tergites.

In posterior abdominal segments, dramatic modulation of *t* expression by temperature is essential for thermal plasticity of pigmentation in females^34^. However, as overexpression at 29 °C of both *y* and *t*, but not the one of *t* alone, is necessary and sufficient to reproduce the black pigmentation phenotype observed at 18 °C, we can conclude that modulation of *y* expression by temperature also contributes to thermal plasticity of pigmentation in the posterior abdominal segments of females.

Our results are remarkably similar to those obtained in the lepidopteran *Junonia coenia*, a species with contrasting seasonal morphs^10^. Spring and autumn morphs have markedly different wing pigmentation patterns. In this butterfly, environmental conditions dramatically modulate the expression of *t* in late pupal stage and *y* in earlier pupal stage. Phenotypic plasticity of pigmentation in insects appears therefore to be a complex process based on transcriptional modulation of multiple pigmentation genes at distinct developmental stages corresponding to their peak of expression. A few of them, such as *t*, play a paramount role whereas others, like *y,* have a more modest contribution.

## Methods

### Fly stocks

We used a *w*^*1118*^ inbred line as wild-type. The *y* allele used was *y*^*1*^. The *UAS-t* line was a gift of Dr Nicolas Gompel. The *y-wing-body-nEGFP* line was from the lab of Sean Carroll. The *pnr-Gal4* (BL3039) and *UAS-y* (BL3043) lines were from the Bloomington stock centre. The *UAS-RNAi-Ddc* (GD3329) line was from the VDRC stock centre (Vienna, Austria).

### Cuticle preparations

Adult flies between 3 and 5 days old were stored for 8 days in ethanol 75% before dissection. Abdominal cuticles were cut just beyond the dorsal midline. After dissection, cuticles were rehydrated in PBS-glycerol for 4 hrs. They were then mounted in Hoyer’s medium.

For nEGFP observations, abdomens were dissected in PBS, fixed 20 minutes in

3.7% paraformaldehyde in PBS, washed twice 10 minutes in PBS and mounted in Mowiol.

### *In situ* hybridization

A 822 bp *y* fragment was amplified by PCR with primers 5′-TGACTTGACCACGGATACGC-3′ and 5′-GGTGGACCCATTGGCAAAAC-3′ from cDNAs previously prepared from pupal abdominal epidermis^34^. This PCR fragment was cloned by Topo-Cloning and LR-Recombination (Gateway) in *pBlueScript* vector (Invitrogen). We used two clones where the *y* fragments were inserted in opposite directions so that sense and antisense Digoxygenin-labeled probes could be synthesized with the same RNA polymerase (T7, Roche) after linearization of the plasmids. *In situ* hybridizations were performed according to the Carroll’s lab protocol (http://carroll.molbio.wisc.edu). Precisely, we followed the protocole “*in situ* hybridization on adult abdomen” modified from the Mark Rebeiz’s one by Héloise D. Dufour. Specificity of the antisense probe was assessed by comparison with signal from the sense probe (Figure S1). Morphological markers (wing color, degree of maturation of abdominal bristles, localization of meconium in the abdomen) were used to stage pharates and collect them at a similar developmental stage whether grown at 18 °C or 29 °C^44^. *In situ* hybridization experiments were performed twice, on individuals grown at 18 °C or 29 °C (between 10 and 20 individuals for each temperature). Staining was stopped after 90 minutes and 80 minutes for the first and the second experiment respectively. Similar results were obtained in each experiment. Representative pictures from the first *in situ* hybridization are shown in Fig. 4.

### Image acquisition

Adult cuticles and abdominal *in situ* hybridizations were imaged with a binocular equipped with Leica DC480 digital camera using the *Leica IM50 Image Manager* software. An annular lamp was used to ensure homogeneous lighting. Care was taken to use identical settings in each set of experiments. The higher magnification pictures showing *y* expression associated with bristles as well as abdominal epidermes of *y-body-nEGFP* pharates were acquired using a micro-apotome (Zeiss). nEGFP was measured in hemi-tergites A5, A6 and A7 using maximum intensity projections of stacks of 14 to 16 pictures (39 to 45 μm thick) using the *Zen* software.

### Statistical analysis

The effect of temperature on *nEGFP* expression in A5, A6 and A7 was tested by a t-test. Homogeneity of variances was previously checked using a Levene test and the appropriate option of the t-test was used (homo- or heteroscedasticity).

## Additional Information

**How to cite this article**: Gibert, J.-M. *et al*. Modulation of *yellow* expression contributes to thermal plasticity of female abdominal pigmentation in *Drosophila melanogaster. Sci. Rep.* **7**, 43370; doi: 10.1038/srep43370 (2017).

**Publisher's note:** Springer Nature remains neutral with regard to jurisdictional claims in published maps and institutional affiliations.

## Supplementary Material

Supplementary Figure S1

## Figures and Tables

**Figure 1:**
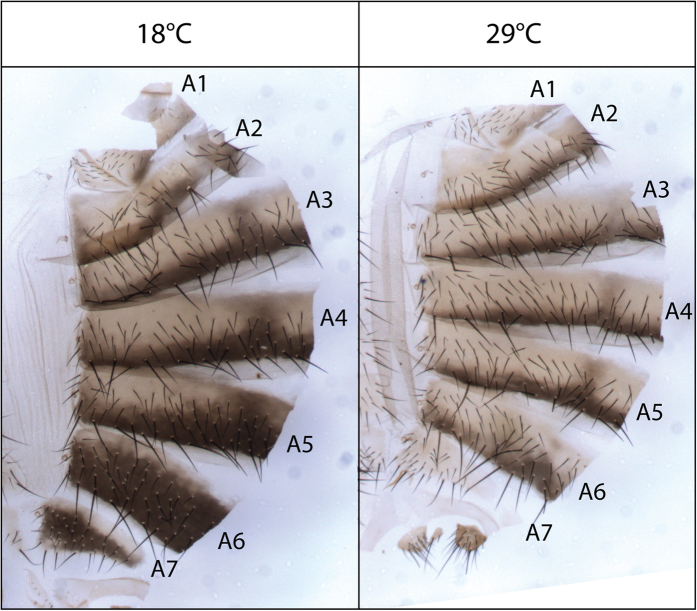
Plasticity of abdominal cuticle pigmentation upon temperature in females. Abdominal cuticles of *w*^*1118*^ females grown at 18 °C or 29 °C. Pigmentation is modulated by temperature in particular in the posterior abdomen (A5, A6 and A7) as previously shown^34^.
