# Supplementary material for: Modulation of yellow expression contributes to thermal plasticity of female abdominal pigmentation in Drosophila melanogaster
Source: Sci Rep. 2017 Feb 23;7:43370. doi: 10.1038/srep43370 (PMC5322495; doi:10.1038/srep43370)
Supplement: Supplementary Figure S1 [file srep43370-s1.doc]

**Figure S1 of:**

**Modulation of *yellow* expression contributes to thermal plasticity of female abdominal pigmentation in *Drosophila melanogaster***

Jean-Michel Gibert*, Emmanuèle Mouchel-Vielh and Frédérique Peronnet


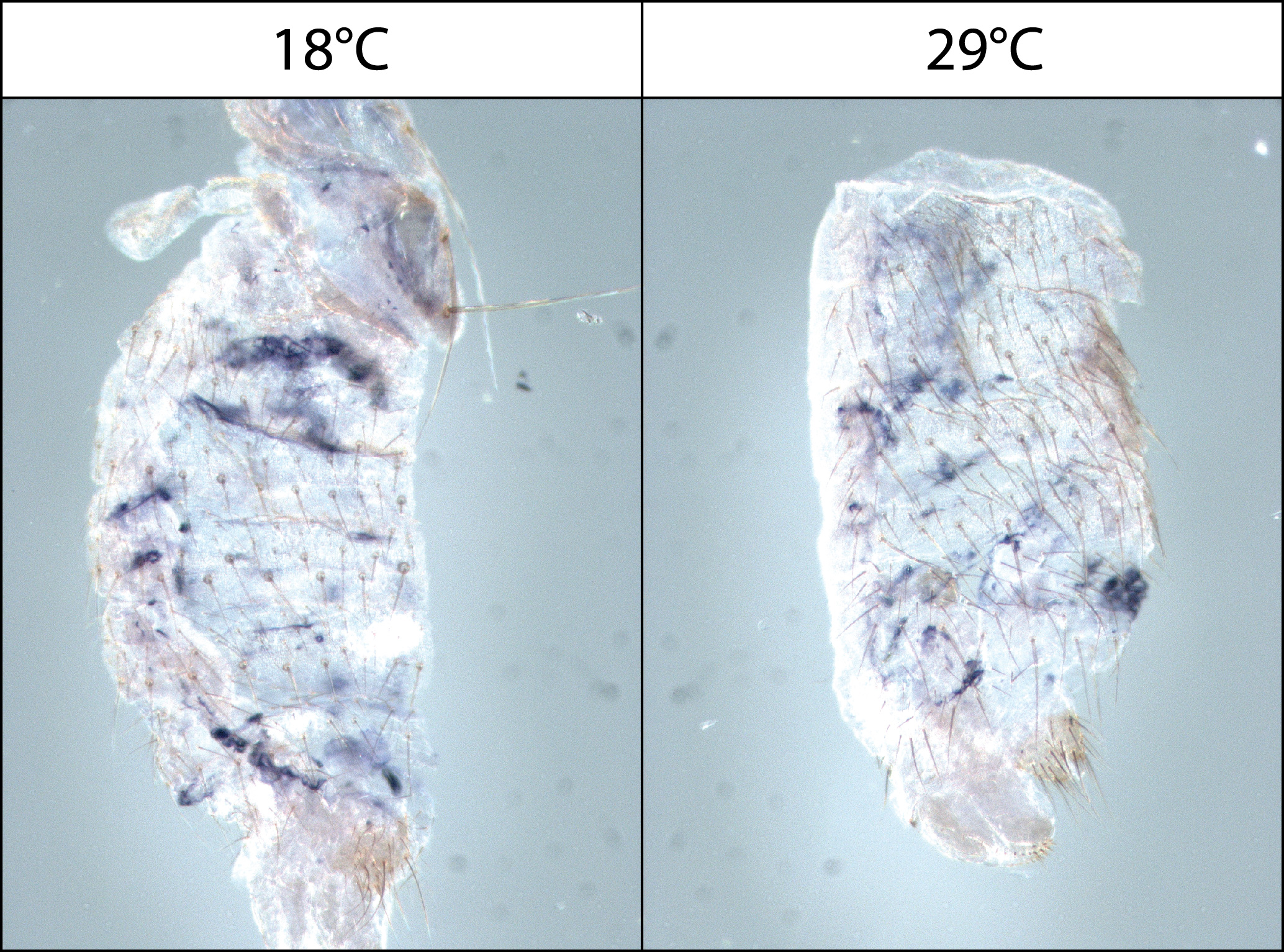


**Figure S1: Control *in situ* hybridization with *y* sense probes.**

No signal is observed in the abdominal epidermis.
